# Pollinator responses to floral colour change, nectar, and scent promote reproductive fitness in *Quisqualis indica* (Combretaceae)

**DOI:** 10.1038/srep24408

**Published:** 2016-04-13

**Authors:** Juan Yan, Gang Wang, Yi Sui, Menglin Wang, Ling Zhang

**Affiliations:** 1 Key Laboratory of Tropical Forest Ecology, Xishuangbanna Tropical Botanical Garden, Chinese Academy of Sciences, Mengla, Yunnan 666303, China; 2 University of Chinese Academy of Sciences, Beijing 100049, China

## Abstract

Floral colour change is visual signals for pollinators to avoid old flowers and increase pollination efficiency. *Quisqualis indica* flowers change colour from white to pink to red may be associated with a shift from moth to butterfly pollination. To test this hypothesis, we investigated *Q. indica* populations in Southwest China. Flowers secreted nectar continuously from the evening of anthesis until the following morning, then decreased gradually with floral colour change. The scent compounds in the three floral colour stages were similar; however, the scent composition was different, and the scent emission rate decreased from the white to red stage. Dichogamy in *Q. indica* prevents self-pollination and interference of male and female functions. Controlled pollinations demonstrated that this species is self-incompatible and needs pollinators for seed production. Different pollinators were attracted in each floral colour stage; mainly moths at night and bees and butterflies during the day. Observations of open-pollinated inflorescences showed that white flowers had a higher fruit set than pink or red flowers, indicating the high contribution of moths to reproductive success. We concluded that the nectar and scent secretion are related to floral colour change in *Q. indica*, in order to attract different pollinators and promote reproductive fitness.

Flower colours act as sensory signals that attract pollinators by ‘advertising’ the quality and quantity of floral rewards^1^,^2^. Floral colour not only differs among flowering plants, but also varies among different populations and individuals of the same species, or even changes during the flower life. Floral colour change is a common phenomenon in angiosperms, occurring in at least 456 species that belong to 268 genera, 78 families, and 33 orders^3^,^4^,^5^. These colour variations are not referred to the darkening or fading of floral senescence, but to changes in fully blooming turgid flowers^3^.

It is well known that pollinators are agents of directional selection on floral colour^6^,^7^,^8^,^9^. Therefore, floral colour changes may act as visual signals for pollinators to avoid old flowers^10^, increase pollinator foraging efficiency, and pollen transfer^11^,^12^. Moreover, flower petals that are no longer receptive functions expand the floral display size and contribute to long-distance signalling to pollinators in order to increase visitation rates^3^,^11^,^13^,^14^,^15^,^16^ and consequently, the reproductive success^12^. In some situations, floral colour change allows pollinators to identify unrewarding flowers at close range, instead of choosing rewarding flowers^14^,^16^,^17^,^18^. This strategy reduces the repeated visits to flowers and increases the pollination efficiency of visitors, preventing long pollinator stays on the same inflorescence, and thus, reducing or preventing geitonogamous pollination^19^,^20^.

However, the relative effectiveness of floral colour change has not been well evaluated with respect to other floral traits, including nectar, pollen, and scent. Scent and floral colour change are all incentives for pollination that often interact. For example, some flowers may have a large amount of nectar as well as high pollen viability and stigma receptivity, but all these characteristics decline after the floral colour change^13^,^14^,^15^,^17^,^18^,^21^,^22^. In other cases, including *Leucojum vernum*, *Nicotiana rustica*, and *Pulmonaria officinalis*, floral colour change ‘advertises’ nectar rewards and successfully attracts pollinators^23^. Previous studies investigated the effects of colour change on the rewards for pollinators and reproductive success; however, little is known about the contribution of different floral colour stages and other characteristics, such as nectar and scent, to pollinator attraction.

*Quisqualis indica* is an Asian tropical climber that undergoes a floral colour change from white to pink to red^24^. Although Eisikowitch and Rotem^25^ described the role of orientation and colour change in pollinator attraction under field conditions in Israel, no detailed studies have been carried out on the pollination biology of this species. Our preliminary field observations indicated that floral colour change is related to increased nectar secretion and a very strong scent. In many cases, flowers change colour, but only attract one group of pollinators^15^,^17^,^18^,^21^; however, the pollination syndromes of this species is assumed to attract two kinds of long tongued insects for pollination 26, moths at night and butterflies during the day. Therefore, floral colour change may be related to a shift from moth to butterfly pollination. To evaluate this assumption, we examined the potential relationship of floral colour change with nectar and scent as well as the resulted changes in pollinator attraction and reproductive success, and aimed to address the following questions: (1) Is the nectar secretion and scent intensity pattern related to the floral colour change? (2) Is the floral colour change related to pollinator shift? and (3) How is pollination achieved, and what is the functional significance of floral colour change?

## Results

### Floral biology and phenology

#### Flowering phenology

In the Xishuangbanna Tropical Botanical Garden (XTBG) and Menglun Nature Reserve (MLD), *Q. indica* flowers opened fairly synchronously on each plant between 19:00 and 20:00. Inflorescences produced a total of 17–44 (mean ± standard error, 30.1 ± 1.2, *N* = 30) flowers that lasted for 5–14 (9.2 ± 0.4, *N* = 30) d. Each day, they produced 1–8 (2.2 ± 0.3, *N* = 30) flowers with 2,640–16,440 (10,253 ± 920, *N* = 30) pollen grains and three ovules per flower. Hence, the mean pollen: ovule ratio (P/O) ratio was 3,418 (Table 1). Flowering occurred mainly from early April to May, whereas sporadic inflorescences were produced until the end of June.

#### Floral colour change

**Figure 1:**
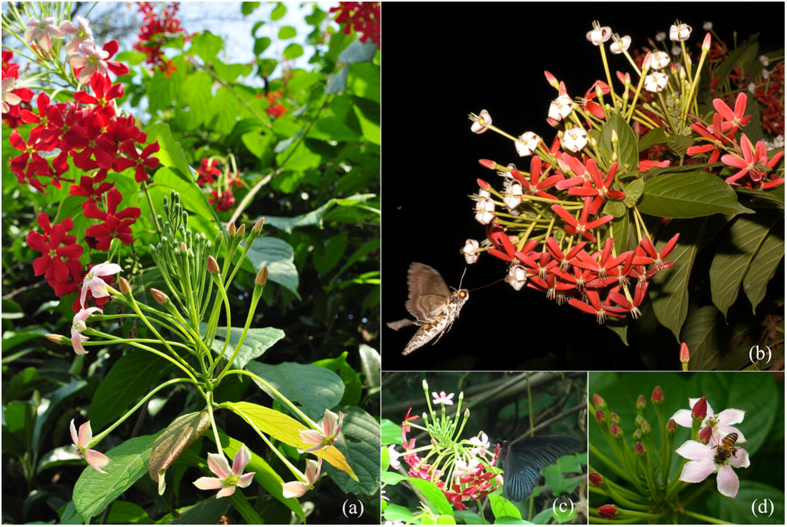
Floral colour change and some visitors in *Quisqualis indica*. (**a**) Blooming inflorescences showing floral colour change from pink to red (Image taken by Mr. Guangyu Liu, Xishuangbanna Tropical Botanical Garden); (**b**) A moth visiting white flowers at night (Image taken by Dr. John Kress, National Museum of Natural History, USA); (**c**) A butterfly visiting pink and red flowers in the day; (**d**) A bee visiting pink flowers.

The petal light reflectance in the white, pink, and red stage (Fig. 1) of *Q. indica* is shown in Fig S1. At anthesis (19:00–20:00), the flowers were white with a round corolla (13.89 ± 0.23 mm in diameter, *N* = 88), which was much smaller than that of the pink (P_1_ < 0.0001) and red (P_2_ < 0.0001) flowers, and had a delicate scent. All flowers were oriented obliquely upward with the petals first deflexed and then expanded. The following day, the flowers turned slightly pink at sunrise, changed to dark pink by midday, whereas after 14:00, they turned red and became pendulous with the corolla reaching the maximum diameter (16.79 ± 0.25 mm, *N* = 88; P _3_ = 0.015). The older red flowers could persist on the inflorescence for 4–6 d.

#### Pollen viability and stigma receptivity

**Figure 2:**
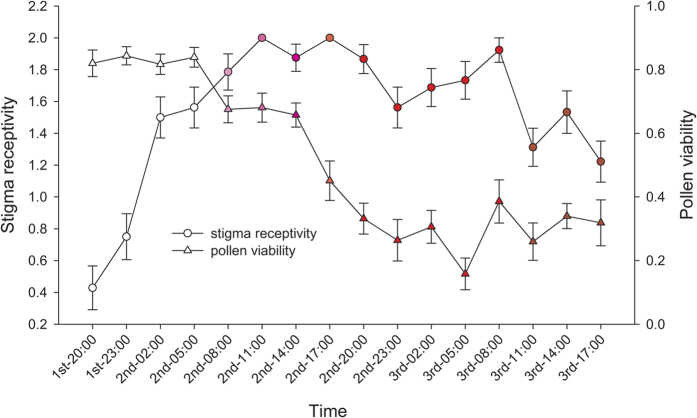
Changes in pollen viability and frequency of stigma receptivity in *Quisqualis indica*. Data are expressed as means ± standard error.

Pollen viability was high at anthesis and remained high (approximately 80%) throughout the white floral stage. Then, it decreased in the pink floral stage by approximately 60%, whereas about 20% of the pollen was still viable in the red floral stage for 2–3 d (Fig. 2). Stigma receptivity was very low at anthesis, increased quickly, reached a maximum at 11:00 the following day in the pink floral stage, and declined gradually at d 3 in the red floral stage (Fig. 2).

#### Floral nectar

**Figure 3:**
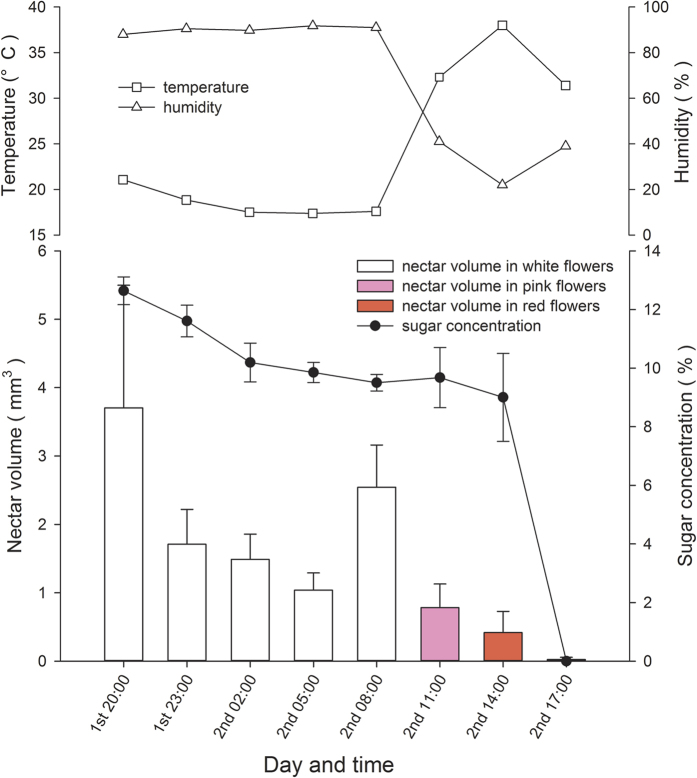
Nectar volume and sucrose concentration of *Quisqualis indica* flowers in relation to temperature and humidity. Data are expressed as means ± standard error.

The flowers produced nectar continuously in the white floral stage from anthesis until 08:00 in the following morning (3.7 ± 1.8 μl, *N* = 15) (Fig. 3). Later, there was a sharp decline in nectar volume, when floral colour changed from pink to red and secretion stopped at approximately 14:00. Concurrently, the nectar sucrose concentration decreased gradually from 12.6 to 9.0 mol L^−1^. Variations in air temperature and relative humidity on flowering days are also shown in Fig. 3.

### Flower visitors and pollinators

**Figure 4:**
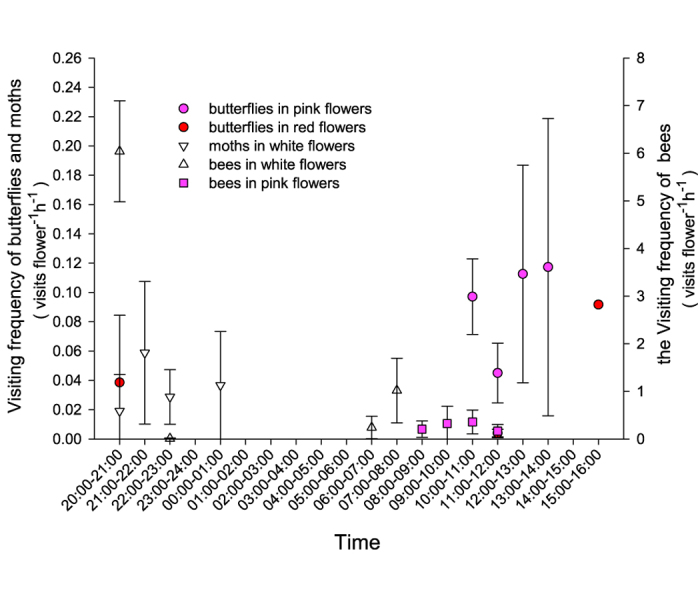
Visiting frequency of pollinators to *Quisqualis indica* flowers at anthesis.

Flowers were visited by bees (mainly *Apis dorsata* and a few *Apis cerana*) in the first hour after anthesis, and then, only hawkmoths (*Macroglossum* sp.) made subsequent visits until the following morning, but with a very low frequency. The highest visitation rates for moths in XTBG were 0.06 ± 0.05 (*N* = 13) visits flower^−1 ^h^−1^ at 21:00–22:00, whereas for bees 1.02 ± 0.68 (*N* = 6) visits flower^−1 ^h^−1^ in the second morning from 07:00–08:00 (Fig. 4). Flowers were visited by two species of butterflies (*Papilio* sp. and *Pieris rapae*) at 10:00–14:00 in the pink floral stage, whereas very few butterflies visited the flowers in the red floral stage. All bees, moths, and butterflies were effective pollinators, because they consistently contacted both anthers and stigmas and had pollen grains deposited on their bodies. Moths visited single flowers to rapidly collect nectar rate and then flew away; butterflies visited single flowers to collect nectar and briefly stayed in an inflorescence; whereas bees visited many newly opened flowers to collect pollen grains and stayed longer in the same inflorescence or different inflorescences within the same plant, which could cause geitonogamy and pollen discounting due to the self-incompatibility in this species.

### Fruit set in different floral colour stages

In the Tongliang, Chongqing (CQTL) population, the fruit set rate of control inflorescences (21.2 ± 10.5%, *N* = 13) was the highest, followed by that of inflorescences exposed to natural pollination in the white stage (15.5 ± 7.1%, *N* = 14), whereas those exposed in the red stage had the lowest fruit set rate (4.2 ± 2.9%, *N* = 14). However, differences in the fruit set rates within the CQTL population were not significant probably because of the small sample sizes and large variation (Fig. S2). In the XTBG population, the fruit set of each treatment was lower than that in the CQTL population. The highest fruit set rate was observed in the white floral stage (8.9 ± 2.2%, *N* = 64), followed by the control (6.5 ± 1.4%, *N* = 30), and both were significantly higher than those in the pink floral stage (1.8 ± 1.2%, *N* = 60; P_1_ = 0.000 and P_2_ = 0.045, respectively) and the red floral stage (0.6 ± 0.6%, *N* = 58; P_3_ = 0.011 and P_4_ = 0.000, respectively) (Fig. S2).

### Controlled pollination experiment

**Figure 5:**
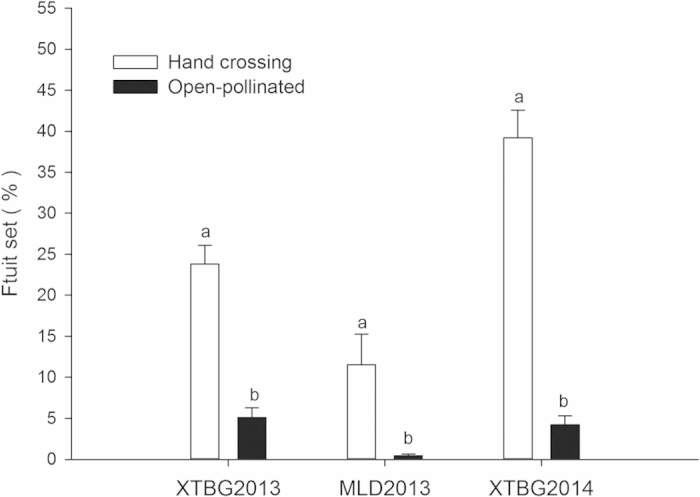
Effects of pollination treatments on fruit set rate in *Quisqualis indica*. Data are expressed as means ± standard error. XTBG, Xishuangbanna Tropical Botanical Garden; MLD, Menglun Nature Reserve.

The fruit set rate in hand cross-pollinations was significantly higher than that in open-pollinated controls in XTBG both in 2013 (11.2 ± 1.8%, *N* = 175 and 5.1 ± 1.1%, *N* = 67, respectively; P_1_ < 0.0001) and 2014 (38.7 ± 3.3%, *N* = 98 and 4.2 ± 1.1%, *N* = 56, respectively; P_2_ < 0.0001), and MLD in 2013 (11.5 ± 3.7%, *N* = 24 and 0.4 ± 0.2%, *N* = 23, respectively; P_3_ = 0.007) (Fig. 5). Hand selfing, hand geitonogamy, and bagging resulted in no fruit set. The number of inflorescences in each treatment was initially around 30 in the MLD population, but samples sizes were reduced due to inclement weather conditions and herbivores.

### Variation in floral scent

**Figure 6:**
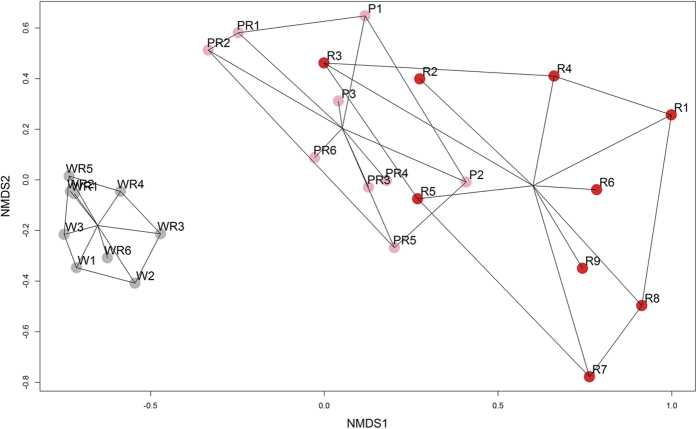
Nonmetric multidimensional scaling (NMDS) ordination of scent profiles emitted by *Quisqualis indica* white flowers (W), white and red flowers (WR), pink flowers (P), pink and red flowers (PR), and red flowers (R) compared with the control (CK) based on Bray-Curtis distance. Data were rotated by principal component to maximize the first dimension. Stress = 0.11.

In total, 35 volatile organic compounds (VOC) were found in the floral scent of *Q. indica*. Twenty-five main compounds, in different ratios, were detected in the white, pink, and red floral stages, including cis-linalool oxide (16.47%, 25.37%, and 7.79%, respectively), epoxylinalool (3.97%, 18.20%, 25.19%, respectively), α-farnesene (2.57%, 9.27%, 7.68%, respectively), and cis-3-hexenyl tiglate (26.03%, 17.29%, 26.45%, respectively). cis-3-Hexenyl butyrate (14.68%), cis-3-hexenyl isovalerate (11.64%), and (Z)-3-hexen-1-ol acetate (7.77%) were mainly identified in the white floral stage (Table 2). The nonmetric multidimensional scaling (NMDS) ordination with Bray-Curtis distances of different odour samples showed great variation in scent composition among the different floral colour stages (Fig. 6). The overall scent profiles were significantly different among the floral colour stages (multiple response permutation procedure [MRPP], A = 0.2584, P = 0.001 between the white and red floral stages; A = 0.2765, P = 0.001 between the white and pink floral stages; and A = 0.1106, P = 0.001 between the pink and red floral stages). The scent profiles of pure white flower clusters and mixed white-red clusters (A = 0.02158, P = 0.3) or those of pure pink clusters and white-pink-red clusters (A = −0.05155, P = 0.707) did not show any significant differences. The scent emitting rate of single white flower (mean ± standard deviation, 3.54 ± 1.28 μg h^−1^) was more than 3-fold higher than that of pink (1.11 ± 0.29 μg h^−1^) or red (0.61 ± 0.11 μg h^−1^) flowers (Fig. S3).

## Discussion

This study revealed that *Q. indica* has an unusual pollination system. Its floral colour change attracts different sets of pollinators, including moths and bees in the white floral stage, bees and butterflies in the pink floral stage, and butterflies in the red floral stage, with all sets facilitating cross-pollination. The flowers secreted nectar continuously and maintained high nectar volumes and sugar concentrations in the white stage, which then decreased gradually from the pink to the red stage; thus, the secretion pattern was related to changes in floral colour. Pollen is viable before stigma receptivity, and this short temporal dichogamy can prevent self-pollination. In this study, the scent profiles changed, and the scent emission rate decreased with the floral colour change from white to pink to red, suggesting some functional relation between scent emission and colour change in order to achieve the best pollination attraction in different floral colour stages. This species is self-incompatible, and the results also showed that fruit set rates were higher when inflorescences were exposed to natural pollination in the white floral stage compared with the pink and red floral stages, suggesting that moths and bees had higher contribution to reproductive success than other insects. Overall, our results indicated that nectar and scent secretion along with floral colour change in *Q. indica* affect pollinator behaviour and promote reproductive fitness.

Floral colour change has been recognized as a special pollination strategy, whereas the overall floral display is enhanced without increasing pollen wastage. The flowers of *Q. indica* are typically pollinated by hawkmoths in the white floral stage^25^,^27^,^28^, in which the large amount of nectar and relatively low sugar concentration meet the known reward levels of the preferred flowers of hawkmoths^28^,^29^. The flowers in the pink and red floral stages become larger and long-tubed and are mainly preferred by butterflies^28^,^30^. In this study, the pollinator composition in the three floral colour stages was different from that described by Eisikowitch and Rotem^25^. According to pollination syndrome concept^26^, *Q. indica* would not be pollinated by short-tongued bees. However, bees visited both white and pink flowers with a quite high frequency, regardless flower orientation, and also multiple flowers within the same inflorescence, probably causing lead to pollen discounting. Moreover, they often visited white flowers before the moths, negatively affecting reproductive success by collecting pollen before the effective pollinators. The synchronous mass flowering in *Q. indica* can cause geitonogamy^31^, which leads to low fruit set rates under natural conditions^32^; however, dichogamy may reduce the risk of geitonogamy and prevent self-pollination^31^.

Floral nectar rewards can affect pollination by manipulating pollinator behaviour^33^, and floral scents are used by flowers to attract pollinators by simultaneously attracting some pollinator taxa while repelling others^34^. In the white stage, the flowers of *Q. indica* secreted nectar and emitted fragrance continuously to attract moths, whereas from the pink to red stage, nectar volume, sugar amount, and scent emission rate reduced considerably, and pollinators shift to bees and butterflies. This study showed that the flowers ‘advertise’ their nectar rewards in the pink and early red floral stages, when pollen viability has declined. These findings were in disagreement with previous studies^14^,^15^,^16^,^17^,^18^,^21^, which showed that flowers changed colour maintaining a relatively high pollen viability and preserving male and female fitness for longer time. Red flowers persist on the inflorescence for 4–6 d, increasing their floral display size and creating a “cloud” of scent, which probably acts as a long distance signal to attract more pollinators^3^,^11^,^13^,^14^,^15^,^16^. Floral nectar secretion and scent changes during floral colour change in *Q. indica* may be part of the flower senescence process. In this study, we could not clarify if nectar and scent changes influence pollinator behaviours separately; thus, further study is needed to determine their individual roles in pollinator attraction.

Floral colour and scent are interlinked traits in plants^35^. Flower scents are classified by their biosynthetic origin into terpenes, phenylpropanoids, and fatty acid derivatives^36^,^37^, and the phenylpropanoid pathway determines floral colour and scent^38^. Some studies demonstrated that these volatiles are derived from two biochemical pathways: one produces acyclic monoterpenes and the oxides, and the other, benzoate and its derivatives^39^. *Q. indica* has a strong floral scent in the white floral stage, when it is pollinated by moths, and then its scent emission rate declines in the pink and red floral stages, when it is pollinated by butterflies. It is possible that the scent contributes more in moth attraction during the night, whereas distinct colours contribute more in bee and butterfly attraction during the day. This transition is consistent with the phenylpropanoid pathway, but light may also be a triggering factor (Zhang *et al*., unpublished data) that stimulates anthocyanin pigment metabolites^40^,^41^,^42^,^43^.

In conclusion, *Q. indica* is a dichogamous and self-incompatible species; therefore, seed production is pollinator dependent. The combination of floral colour change, nectar secretion, and scent releasing pattern in *Q. indica* probably influence pollinator behaviours and plant reproductive success. Additionally, floral colour change may be a type of adaption to pollinator shift from nocturnal to diurnal. All sets of pollinators play a role in reproductive success, but pollinators in the white floral stage contribute more to fruit production than those in other stages, suggesting that diurnal pollination is a form of compensation, in cases that nocturnal pollination is not successful. Overall, our results indicated that nectar and scent secretion patterns reflect the floral colour change rhythm in *Q. indica*, and multiple signals attract and manipulate pollinators in order to promote reproductive fitness. However, further research is needed to better define the interactions between two key floral traits, colour change and scent.

## Methods

### Study species and sites

*Q. indica* Linn (Combretaceae) is an Asian tropical climber that is mainly distributed in southwest China, including Sichuan, Yunnan, Guizhou, Hunan, Guangxi, and Guangdong^24^. The plant is also cultivated in China as an ornamental, and its seeds are used medicinally to kill intestinal parasites. In Yunnan Province, the region in which our studies were conducted, *Q. indica* flowers from April until June. The majority of field observations and experiments on *Q. indica* was conducted at two locations over a three-year period (2012–2014). The first site was in a liana collection of XTBG (21°45′N, 101°02′E; 580 m above sea level), dominated by *Lagerstroemia tomentosa* (Lythraceae) and *Ficus callosa* (Moraceae). *Q. indica* was cultivated for many years at this site and also naturalized into the adjacent limestone forest (semi-natural habitat). The second site was approximately 10 km away in MLD that belongs to Menglun Nature Reserve (natural habitat) (21°55′N, 101°19′E; 590 m above sea level) that is dominated by *Ficus langkongensis* (Moraceae). We also conducted phenological observations and experiments in CQTL (29°50′N, 106°03′E; 400 m above sea level), which is known for the cultivation of *Q. indica* for Chinese medicine (cultivation habitat).

### Floral biology and phenology

Preliminary phenological observations in natural populations as well as the flowering and fruit set monitoring of cultivated plants were conducted in XTBG in 2012–2014. We randomly selected 30 inflorescences (1–3 per plant) in four study plots and recorded the total number of flowers and duration of flowering. Additionally, we estimated the number of pollen grains and ovules in one flower per inflorescence. We used a haemocytometer to estimate pollen production per flower as described by Dafni^44^. We used pollen and ovule numbers to calculate the mean P/O of the flowers. To investigate floral colour change, we monitored five flowers from different inflorescences every 2 h for 2 d and recorded the time and flower colour, which was assessed visually and then measured with a spectrophotometer (Ocean Optics, USA). In addition, we also measured the petal length of three flowers from each inflorescence of 30 plants in the white, pink, and red floral stage using vernier calipers (0.01 mm; Guanglu, China) to determine any changes in the corolla size.

We used 0.1% 3-(4,5-dimethylthiazol-2-yl)-2,5-diphenyl-tetrazolium bromide to test the presence of dehydrogenase as an assay for pollen viability^45^. A total of 64 flowers from four study plots were covered using nylon mesh bags prior to anthesis to prevent insect visits, and then four flowers were used to test pollen viability every 3 h from anthesis to flower wilting. The same assay was used to assess stigma receptivity^45^, using the same number of experimental flowers from the same plants. All tests were carried out under favourable weather conditions.

Every day, we randomly selected three inflorescences from each of three study plants located approximately 500 m apart and covered them with nylon net bags in the afternoon prior to anthesis. Three flowers in each inflorescence were used to measure nectar secretion every 3 h from 20:00 to 17:00 the following day. The nectar volume was measured with 10 and 20 μl ‘micro-cap’ calibrated capillary tubes (Sigma-Aldrich, USA), and nectar sucrose concentration with a hand-held, temperature-compensated refractometer (Bellingham + Stanley Ltd., UK). This experiment was repeated for 3 d (10–12 April 2012), and nine inflorescences with a total of 27 flowers were evaluated. At the same time, the temperature and humidity were measured with a hygrothermograph (0.1 °C, Wangyunshan-Fuzhou, China).

### Flower visitors and pollinators

We observed flower visitors to *Q. indica* in 2012 and 2013 in MLD and XTBG for a total of 81 h and 144 h, respectively, under favourable weather conditions. Observations were made continuously from 20:00 (beginning of anthesis) to 8:00 the following morning by video recording during the night and visually during the day. We recorded the total number of visitors per individual flower and the number of flowers visited by each kind of insect. The visiting frequency of each kind of insects was calculated as the number of visits per flower per hour. We assigned insects as visitors or pollinators, based on their behaviour and likelihood of mediating pollination. Visitors were insects observed on inflorescences, whereas pollinators were insects that consistently contacted both anthers and stigmas and had pollen grains deposited on their bodies. We photographed every type of flower visitor, and voucher specimens of insects were preserved in the insect collections of XTBG.

### Field manipulative experiment

To investigate the reproductive contribution of different floral colour stages, we conducted a manipulative field experiment in the XTBG and CQTL populations using inflorescences exposed to natural pollination. We set up four treatments with 30 plants per treatment using 1–3 randomly chosen inflorescences per plant. All inflorescences were covered with nylon net bags to prevent insect visits prior to treatment. The treatments included inflorescences exposed to natural pollination in (1) the white floral stage, (2) the pink floral stage, (3) the red floral stage, and (4) all floral colour stages (control). Two months later, when fruits were mature, we counted the fruit set of inflorescences.

### Controlled pollination experiment

We performed five pollination treatments on 1–3 inflorescences of 30 randomly selected *Q. indica* plants in the XTBG and MLD populations in 2013 and 2014 to examine the capacity for autonomous self-pollination and evaluate the importance of insect visitors. The treatments were: (1) open-pollination (control), (2) hand selfing, (3) hand outcrossing, (4) hand geitonogamous pollination, and (5) bagging (autonomous self-pollination). For each treatment, except for the control, we covered inflorescences with bags prior to anthesis to prevent pollinator access, and the flowers were emasculated to prevent self-pollination. We performed hand cross-pollinations using pollen from plants up to 500 m away and randomly selected from 10 inflorescences. Not all flowers within an inflorescence were cross-pollinated because of the technical difficulties related to bud emasculation in this species.

### Collection and identification of floral scents

Floral scents were collected *in situ* from flowers in the white, pink, and red stage from four *Q. indica* plants using the dynamic headspace adsorption method^46^, and also included inflorescences with a combination of white and red flowers or pink and red flowers. For each collection, an inflorescence with 5–25 flowers was covered with mesh bags to prevent pollinator access. Some leaves close to the inflorescence were removed from the branch at least 24 h prior to the scent collection experiment. For each sample, the average number of flowers used in the white, pink, and red stage was 16, 16, and 15.3, respectively. We enclosed a whole inflorescence within an odourless polyethylene terephthalate bag (Kalle Nalo GmbH, Germany) for the volatile collection. Airflow was maintained through the bag by a battery-driven air pump (Qihai Machinery and Electric Co., China). The air was purified by active charcoal, introduced into the bag at a flow rate of 400 ml min^−1^, pumped out of the bag through a glass cartridge (7 mm internal diameter), and filled with 300 mg of Super Q adsorbent (80–100 mesh size; ARS Inc., USA) at a flow rate of 300 ml min^−1^. The adsorbent filters were eluted three times with 100 μl of hexane. To detect any environmental contamination during the volatile collections, ambient air was collected at the same place using the same dynamic headspace technique. The leaf branch was used as control in some cases that only a few leaves were included in the inflorescent treatment. The collection was carried out for approximately 3 h in each floral colour stage. Any volatile compounds that were common with the control treatment were removed before the analysis. To calculate the absolute amount of floral volatiles, two internal standards, octane and decyl acetate, were added to each sample as described by Chen *et al*.^46^. The extract samples were then stored at −20 °C until analysis.

The extracts were analysed using a coupled gas chromatography mass spectrometer (GC-MS) system (Agilent, USA) equipped with an HP-5MS column (30 m × 250 μm × 0.25 μm). Helium was the carrier gas, and ionization occurred by electron impact (70 eV; source temperature 230 °C). For each sample, 1 μl was injected at an injector temperature of 250 °C. The initial column temperature was 40 °C, and then increased by 3 °C min^−1^ up to 100 °C, by 5 °C min^−1^ up to 200 °C, and finally by 20 °C min^−1^ up to 250 °C, which was maintained for 10 min. Compound identification was based on matching mass spectra in the NIST08 Mass Spec library and confirmed by the retention index (RI) in the NIST online library (http://webbook.nist.gov). The absolute values of all compounds were estimated using the average peak area of the two internal standards as a reference scale. The relative proportions of each compound were also calculated.

### Data analysis

To assess the differences of corolla diameter between floral colour stages, we carried out one-way analysis of variance (ANOVA) in conjunction with Fisher’s least significant difference test to identify pairwise differences. The mean fruit set was calculated in all treatments before statistical analysis. We used Kruskal-Wallis test to identify differences in fruit set rates among different treatments in XTBG and CQTL in 2013 and independent-sample t test to identify differences in fruit set under different pollination treatments in XTBG and MLD in 2013 and XTBG in 2014. All analyses were performed using SPSS 20.0 (IBM, USA). A matrix of the relative proportions of all the detected compounds in the three floral colour stages was used to conduct multivariate analysis in R 3.0.0 (https://www.r-project.org/) with the ‘vegan’ package^47^. Scent composition among different floral colour stages was also compared. MRPP was performed based on the matrix of mean dissimilarities with 999 permutations to test the null hypothesis (scent profiles among floral colour stages had no differences). NMDS was used to find the best two-dimensional representation of the distance matrix. To evaluate each configurations that produces the distance matrix, we tested different stress values. The fit of the produced distance matrix to the observations increased with the decreasing stress values. These analyses were repeated until two similar configurations with minimum stress values were obtained.

## Additional Information

**How to cite this article**: Yan, J. *et al*. Pollinator responses to floral colour change, nectar, and scent promote reproductive fitness in *Quisqualis indica* (Combretaceae). *Sci. Rep*. **6**, 24408; doi: 10.1038/srep24408 (2016).

## Supplementary Material

Supplementary Information

## Figures and Tables

**Table 1:** Floral characters of *Quisqualis indica* in the Xishuangbanna Tropical Botanical Garden population.

	Diameter in length			No. of flowers/inflorescence	Duration of an inflorescence (d)	No. of flowers/(day inflorescence)	Pollen grains	Ovules	P/O ratio
	White flowers	Pink flowers	Red flowers						
Mean ± SE	13.89 ± 0.22	15.98 ± 0.2	16.82 ± 0.25	30.1 ± 1.2	9.2 ± 0.4	2.2 ± 0.3	10253 ± 920	3	3418
Range	10.55–20.75	11.33–20.01	12.87–24.69	17–44	5–14	1–8	2640–16440	3	-
n	88	88	88	30	30	30	30	30	30

P/O, pollen: ovule.

**Table 2:** Occurrence (OCC) and relative abundance (Mean ± standard deviation) of volatile compounds emitted by *Quisqualis indica* in different floral colour stages.

Compounds	Retention index	White inflorescences		Pink inflorescences		Red inflorescences	
		OCC	MEAN ± SD	OCC	MEAN ± SD	OCC	MEAN ± SD
cis-3-Hexenol	859	9	2.47 ± 1.04	0	–	0	–
Methyl tiglate	864	6	0.21 ± 0.25	8	2.77 ± 3.62	8	12.04 ± 13.50
α-Methyl- methylbutanoate	783	6	0.06 ± 0.06	0	–	1	0.15 ± 0.44
β-Myrcene	992	4	0.02 ± 0.02	3	0.08 ± 0.13	0	–
cis-3-Hexen-1-ol, acetate	1007	9	7.77 ± 2.97	3	0.11 ± 0.18	2	0.34 ± 0.83
Acetic acid, hexyl ester	1014	9	0.70 ± 0.31	0	–	0	–
5-Methyl-3-(1-methylethylidene)- 1,4-hexadiene	1023	3	0.03 ± 0.04	3	0.14 ± 0.23	1	0.06 ± 0.17
trans-β-Ocimene	1050	9	0.58 ± 0.25	9	3.30 ± 2.06	8	2.38 ± 1.86
cis-β-Ocimene	1039	9	0.33 ± 0.08	9	1.53 ± 1.57	2	0.15 ± 0.34
cis-Linalool oxide	1061	9	16.47 ± 3.52	9	25.37 ± 9.85	9	7.79 ± 2.94
Benzoic acid, methyl ester	1088	3	0.06 ± 0.09	6	0.38 ± 0.32	1	0.09 ± 0.27
Linalool	1097	9	5.63 ± 4.17	9	6.46 ± 3.22	6	2.07 ± 2.19
6-Ethenyldihydro-2,2,6-trimethyl-2H-pyran-3(4H)-one	1107	9	0.34 ± 0.17	7	0.86 ± 0.61	2	0.24 ± 0.50
Neo-allo-ocimene	1129	9	0.40 ± 0.22	9	2.22 ± 2.04	4	1.15 ± 1.62
Benzyl nitrile	1143	9	4.81 ± 1.67	8	1.63 ± 1.24	3	0.38 ± 0.86
Epoxylinalol	1173	9	3.97 ± 1.79	9	18.20 ± 8.26	9	25.19 ± 11.59
cis-3-Hexenyl butyrate	1197	9	14.68 ± 3.41	5	0.32 ± 0.36	1	0.07 ± 0.22
cis-3-Hexenyl isovalerate	1230	9	11.64 ± 2.78	8	0.63 ± 0.36	1	0.05 ± 0.15
Indole	1287	6	0.82 ± 1.84	1	–	0	–
cis-3-hexenyl tiglate	1320	9	26.03 ± 7.35	9	17.29 ± 8.92	9	26.45 ± 15.73
α-Copaene	1377	6	0.10 ± 0.09	8	4.01 ± 4.85	7	9.25 ± 13.43
cis-3-Hexenyl hexanoate	1380	9	0.54 ± 0.18	1	0.05 ± 0.14	0	–
β-Cuvebene	1387	4	0.02 ± 0.03	6	0.66 ± 0.86	5	1.46 ± 2.28
Tetradecane	1400	0	–	4	0.10 ± 0.16	1	0.04 ± 0.13
1-Isoquinolinecarbonitrile	1442	6	0.11 ± 0.13	4	0.72 ± 0.97	3	0.45 ± 0.69
α-Caryophyllene	1449	1	–	2	0.12 ± 0.23	1	0.02 ± 0.07
Alloaromadendrene	1464	0	–	3	0.19 ± 0.30	0	–
Germacrene D	1486	0	–	3	0.16 ± 0.26	1	0.19 ± 0.56
cis,trans-α-Farnesene	1491	4	0.17 ± 0.25	4	0.42 ± 0.67	2	0.30 ± 0.60
Jasminlactone	1595	4	0.04 ± 0.05	2	0.19 ± 0.38	2	0.15 ± 0.29
α-Farnesene	1509	9	2.57 ± 3.41	9	9.27 ± 6.62	9	7.68 ± 5.24
δ-Cadinene	1526	2	0.02 ± 0.03	4	0.47 ± 0.63	4	1.13 ± 1.78
cis-3-Hexenyl benzoate	1571	7	0.29 ± 0.34	1	0.02 ± 0.06	0	–
2-Phenylethyl tiglate	1584	2	0.01 ± 0.02	4	0.66 ± 0.82	2	0.29 ± 0.58
Methyl hexadecanoate	1920	4	0.31 ± 0.57	5	1.68 ± 2.11	1	0.48 ± 1.43
