# Supplementary material for: Pollinator responses to floral colour change, nectar, and scent promote reproductive fitness in Quisqualis indica (Combretaceae)
Source: Sci Rep. 2016 Apr 13;6:24408. doi: 10.1038/srep24408 (PMC4829896; doi:10.1038/srep24408)
Supplement: Supplementary Information [file srep24408-s1.pdf]

Pollinator responses to floral colour change, nectar, and scent promote reproductive fitness of *Quisqualis indica* (Combretaceae)

Juan Yan[1,2], Gang Wang[1], Yi Sui[1,2], Menglin Wang[1,2], Ling Zhang[1,*]

[1]Key Laboratory of Tropical Forest Ecology, Xishuangbanna Tropical Botanical Garden, Chinese Academy of Sciences, Mengla, Yunnan 666303, China

[2]University of Chinese Academy of Sciences, Beijing 100049, China

[*] Author for correspondence: Ling Zhang

Figure legends

Figure S1. Reflected light rate of *Quisqualis indica* petals in different floral colour stages.

Figure S2. Effects of treatments on fruit set rate in different floral colour stages of the Tongliang, Chongqing (CQTL), and Xishuangbanna Tropical Botanical Garden *Quisqualis indica* populations. Sample sizes are marked in each bar. Data are expressed as means ± standard error. Different letters indicate significant differences at $P < 0.05$.

Figure S3. Differences in the scent emission of *Quisqualis indica* flowers in different floral colour stages.

Figure S1.

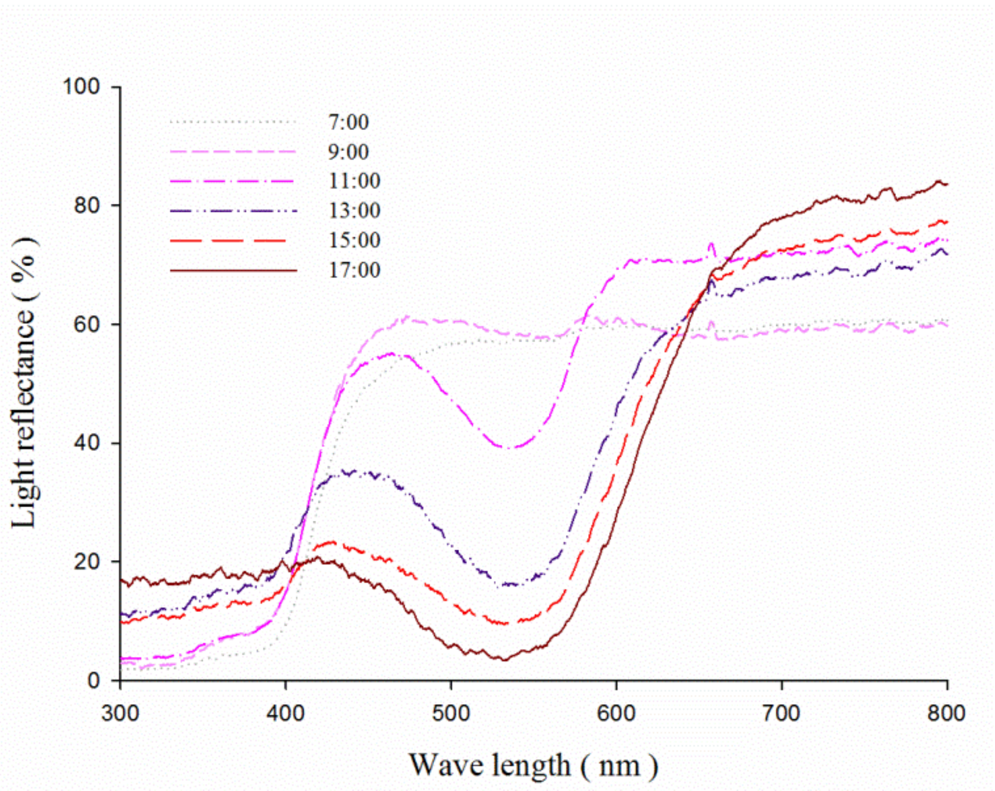
7:00
9:00
11:00
13:00
15:00
17:00
Light reflectance (%)
Wave length (nm)
0
20
40
60
80
100
300
400
500
600
700
800

Figure S2.

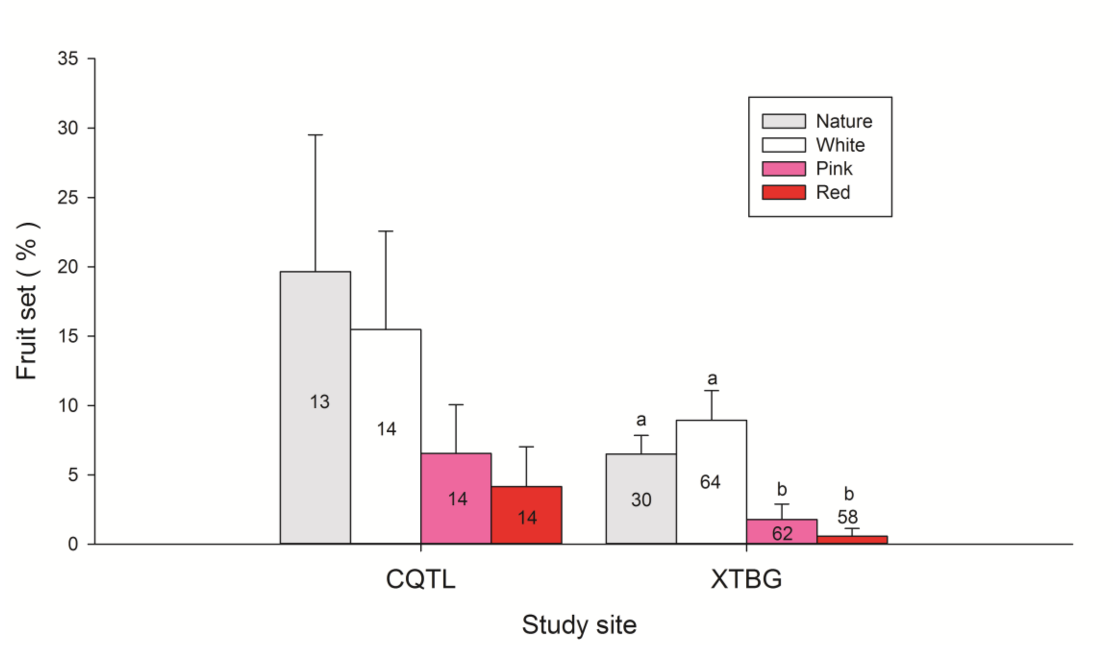

35
30
25
20
15
10
5
0
Fruit set (%)
Nature
White
Pink
Red
13
14
14
14
a
a
30
64
b
b
62
58
CQTL
XTBG
Study site

Figure S3.

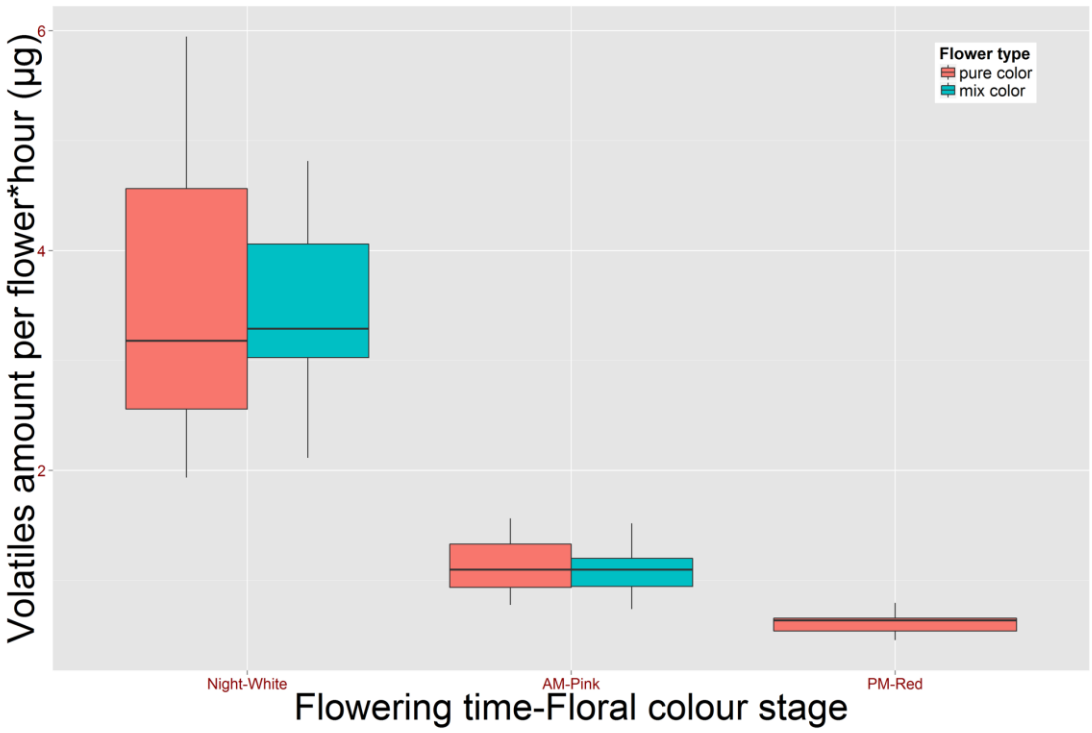
Flower type
pure color
mix color
6
4
2
Volatiles amount per flower*hour (µg)
Night-White
AM-Pink
PM-Red
Flowering time-Floral colour stage
